# Application of the FRAME-IS to a multifaceted implementation strategy

**DOI:** 10.1186/s12913-024-11139-0

**Published:** 2024-06-01

**Authors:** Antoinette Schoenthaler, Franze De La Calle, Elaine De Leon, Masiel Garcia, Doreen Colella, Jacalyn Nay, Isaac Dapkins

**Affiliations:** 1 Institute for Excellence in Health Equity, NYU Langone Health, 180 Madison Avenue, 752, New York, NY 10016 USA; 2 Department of Medicine, NYU Grossman School of Medicine, New York, NY 10016 USA; 3 Family Health Centers at NYU Langone Health, Brooklyn, NY 11209 USA

**Keywords:** Adaptation, Implementation strategy, Practice facilitation, Federally qualified health center

## Abstract

**Background:**

Research demonstrates the importance of documenting adaptations to implementation strategies that support integration of evidence-based interventions into practice. While studies have utilized the FRAME-IS [Framework for Reporting Adaptations and Modifications for Implementation Strategies] to collect structured adaptation data, they are limited by a focus on discrete implementation strategies (e.g., training), which do not reflect the complexity of multifaceted strategies like practice facilitation. In this paper, we apply the FRAME-IS to our trial evaluating the effectiveness of PF on implementation fidelity of an evidence-based technology-facilitated team care model for improved hypertension control within a federally qualified health center (FQHC).

**Methods:**

Three data sources are used to document adaptations: (1) implementation committee meeting minutes, (2) narrative reports completed by practice facilitators, and (3) structured notes captured on root cause analysis and Plan-Do-Study-Act worksheets. Text was extracted from the data sources according to the FRAME-IS modules and inputted into a master matrix for content analysis by two authors; a third author conducted member checking and code validation.

**Results:**

We modified the FRAME-IS to include part 2 of module 2 (what is modified) to add greater detail of the modified strategy, and a numbering system to track adaptations across the modules. This resulted in identification of 27 adaptations, of which 88.9% focused on supporting practices in identifying eligible patients and referring them to the intervention. About half (52.9%) of the adaptations were made to modify the context of the PF strategy to include a group-based format, add community health workers to the strategy, and to shift the implementation target to nurses. The adaptations were often widespread (83.9%), affecting all practices within the FQHC. While most adaptations were reactive (84.6%), they resulted from a systematic process of reviewing data captured by multiple sources. All adaptations included the FQHC in the decision-making process.

**Conclusion:**

With modifications, we demonstrate the ability to document our adaptation data across the FRAME-IS modules, attesting to its applicability and value for a range of implementation strategies. Based on our experiences, we recommend refinement of tracking systems to support more nimble and practical documentation of iterative, ongoing, and multifaceted adaptations.

**Trial registration:**

Clinicaltrials.gov NCT03713515, Registration date: October 19, 2018.

**Supplementary Information:**

The online version contains supplementary material available at 10.1186/s12913-024-11139-0.

## Contributions to the literature

- Research has shown that tools like the Framework for Reporting Adaptations and Modifications to Evidence-based Implementation Strategies (FRAME-IS) are beneficial for documenting changes to implementation strategies that support integration of evidence-based interventions into practice.
- Although a growing number of studies have used the FRAME-IS to document and describe adaptations, they mainly focus on distinct implementation strategies like training. There is little data to show the utility of FRAME-IS for multifaceted strategies like practice facilitation.
- This study contributes to the literature by demonstrating the applicability and value of FRAME-IS for documenting adaptations to multifaceted implementation strategies, when modified for that purpose.

## Background

Growing evidence demonstrating the importance of improving the *fit* of an evidence-based intervention to the setting in which it is implemented has catalyzed research to identify methods to document and track adaptations [1]. Adaptations are defined as changes or modifications to the design or delivery of interventions to improve their fit within a given practice context and population [2, 3]. Previous studies have shown that planned adaptations to improve intervention fit are multidimensional and occur throughout the implementation process, without compromising the level of fidelity [1, 4, 5]. Recently, implementation science research has extended the concept of adaptation beyond tracking changes to the intervention itself to also consider the implementation strategies that support its integration into practice [6–9]. Frameworks such as the FRAME-IS [Framework for Reporting Adaptations and Modifications for Implementation Strategies] have been developed to facilitate the collection of structured data to document and describe adaptations to implementation strategies [6].

The FRAME-IS was developed to facilitate the collection of structured data to describe adaptations to implementation strategies. The tool uses a modular framework to guide practitioners and researchers in documenting decisions around *what, when, how, and why* to modify an implementation strategy supporting the evidence-based intervention. The utility of documenting and understanding adaptations to implementation strategies is particularly critical for safety net settings such as federally qualified health centers (FQHC) that often possess unique financial and administrative threats to intervention fit [10]. FQHCs, which primarily care for medically underserved communities, are often faced with limited resources and staffing and are subject to lower reimbursement rates for healthcare services [11, 12]. These complexities require the adaptation of implementation strategies to maximize intervention fit to the outer (e.g., reimbursement policies) and inner (e.g., organizational culture, implementation climate) practice context.

A small but growing number of studies have utilized the FRAME-IS to provide important information on the adaptability of various implementation strategies in diverse settings including FQHCs [6, 13–16]. However, most of these studies are limited by their focus on a discrete implementation strategy (e.g., training), which do not reflect the complexity of multifaceted strategies such as practice facilitation (PF). To address this limitation, we leverage our ongoing trial, which is evaluating the effectiveness of PF on implementation fidelity of an evidence-based technology facilitated team care model within a large FQHC, to achieve two goals: (1) to conduct an ancillary study that characterizes adaptations to our implementation strategy using the FRAME-IS and (2) report on our experience of using the FRAME-IS in the context of a complex practice-based intervention and provide lessons learned for future application.

## Methods

### Setting and study context

#### Overview of the *a*dvancing *l*ong-term improvements in hypertension Outcomes through a *t*eam-based care *a*pproach (ALTA) trial

We are currently conducting a stepped-wedge cluster randomized controlled trial using a strategy to implement remote patient monitoring (RPM) supported by team-based care [herein called ALTA] in 700 patients with uncontrolled hypertension (HTN) receiving care at a large FQHC (clinicaltrials.gov NCT03713515) [17]. The FQHC where ALTA is being implemented provides healthcare services in southwest and central Brooklyn, capturing a diverse population of mostly Hispanic and Black immigrants (69.6%) facing poverty, and cultural, as well as language barriers (41% are best served in a language other than English).

The trial is being conducted in two phases: a pre-implementation phase where qualitative interviews, workflow analyses and survey data are used to refine the PF strategy, based on the Consolidated Framework for Implementation Research [18] and a 12-month implementation phase, guided by Proctor’s Implementation Outcomes Framework [19]. The primary outcome of the trial is implementation fidelity, which is defined as the degree to which: (1) the implementors (in this case clinic providers and staff) adhere to the ALTA intervention protocol, (2) the dose of ALTA delivered, (3) quality of delivery by the implementors, (4) ALTA component differentiation (i.e., as compared to other practice initiatives), (5) patient exposure to ALTA, and (6) patient and FQHC responsiveness (i.e., satisfaction) to ALTA [17, 19].

**Fig. 1:**
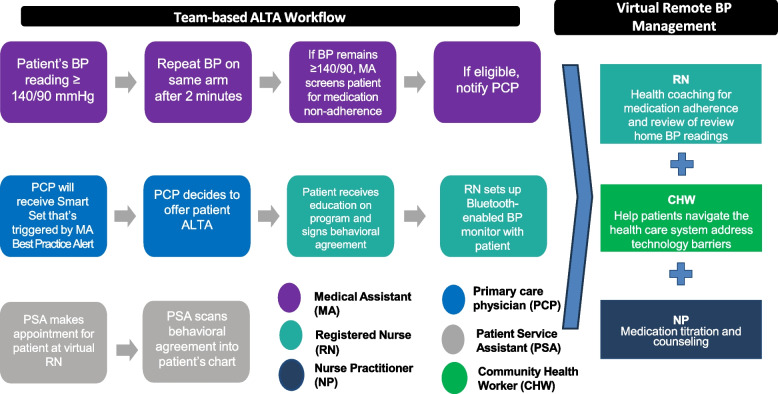
ALTA Team-based Intervention

ALTA is an evidence-based intervention that utilizes a RPM platform that is fully integrated into the electronic health record (EHR) in combination with best practices from the Target:BP MAP [Measure Accurately, Act Rapidly, Partner with Patients] protocol to provide practices with a standardized strategy to improve medication adherence and blood pressure (BP) control (Fig. 1) [20]. ALTA consists of five components: (1) identifying patients with uncontrolled HTN and screening them for medication non-adherence using standard protocols and tools embedded in the RPM platform (*Measure Accurately*); (2) referring patients to a nurse for training in RPM, accurate self BP measurement, and enrollment in health coaching; (3) coaching conducted by a centralized virtual team of registered nurses and nurse practitioners using structured tools in the RPM platform to discuss patients’ home BP readings; establish treatment goals; identify barriers and facilitators to medication adherence; develop patient-centered goals and action plans; and use a structured treatment algorithm to optimize patient antihypertensive medication regimen (*Partner with Patients)*; (4) documenting progress notes in the RPM platform to inform the care team of the patient’s action plans and any changes to the antihypertensive medication regimen; and (5) monitoring patient home BP data and scheduling follow-up sessions for coaching, BP checks, and medication adjustments (*Act Rapidly*).

The PF strategy is designed to stimulate specific, actionable steps that the practices can undertake to build an internal foundation that supports the implementation of ALTA into standard care [17, 21, 22]. As shown in Table 1, research-supported practice facilitators employ a suite of techniques, informed by the ERIC [Expert Recommendations for Implementing Change] compilation to foster collaborative team-based problem solving, build effective team communication, leverage data and health information technology to drive improvement, establish and share common goals between the facilitators and the implementors, and develop skills in continuous quality improvement methods, such as Plan Do Study Act (PDSA) cycles [23]. For the first three months of implementation, the practice facilitators meet biweekly with the practice providers and staff to review their progress, discuss barriers to reach practice goals, co-develop strategies and tools to support implementation, and review metrics that help to monitor how well ALTA is being implemented (e.g., reviewing the EHR to identify patients who did not have an RPM order). In the remaining nine months, onsite meetings are held monthly with additional communication by phone or email, as needed. The focus of the current manuscript is to characterize adaptations to the PF strategy supporting the implementation of ALTA into standard care at the FQHC.

**Table 1:** Description of original ALTA practice facilitation implementation strategy

ALTA Intervention Component	Implementation Strategy Description	ERIC Category
**Identify**	Provide training in proper blood pressure measurement protocol to assist practice staff in identifying ALTA-eligible patients	Conduct ongoing training
	Conduct PDSA cycles to assist practices in implementing screening criteria to identify ALTA-eligible patients	Conduct cyclical small tests of change
	Provide technical assistance in the use of EHR and patient portal to identify ALTA-eligible patients	Provide ongoing consultation
	Develop systems to monitor enrollment of ALTA-eligible patients	Develop and organize quality monitoring systems
**Refer**	Assist practice staff in developing a workflow that supports referral to a health coach	Workflow assessment and testing
	Assist practice staff in creating a system to identify and act on missed opportunities for referral to a health coach	Audit and provide feedback
	Assist practice staff in onboarding ALTA-eligible patients for RPM	Provide ongoing consultation (Staff facing) Purposely reexamine the implementation (Patient facing)
**Coach**	Assist practice staff in using patient-centered communication skills	Training
**Monitor**	Assist practice staff in identifying opportunities for follow-up	Audit and provide feedback
**Document**	Assist practice staff in allocating resources for quality improvement activities and developing a reporting process	Develop and organize quality monitoring systems

### Data collection

The structured forms to track adaptations according to the FRAME-IS were developed a priori during the initial study start-up period. Three data sources were used to document and characterize adaptations to the PF strategy: (1) meeting minutes of the research and FQHC implementation committee, (2) narrative reports completed by the practice facilitators, and (3) structured notes captured on root cause analysis and PDSA worksheets. This data was collected on an ongoing basis throughout the pre-implementation and implementation phases. However, the research team coded the data retrospectively, once the phases ended at each site. Verbal consent was obtained at the practice level from the medical director and practice staff at each primary care practice prior to any data collection during the implementation phase of the trial. A waiver of consent from the institutional review board at NYU Langone Health (#18–01290) was obtained for any patients included in the trial.

#### Implementation committee team meetings

Since the beginning of the trial, members of the research and FQHC teams including the project principal investigator, practice facilitators, FQHC leadership, senior practice management, provider champions, and quality improvement team have met biweekly to discuss ALTA implementation at each of the practices. Topics covered include reviewing EHR data on the uptake of ALTA at each of the practices (e.g., whether patients were screened for medication non-adherence, referred for RPM, met with the nurse for onboarding and RPM education), discussing barriers and facilitators to implementation, and identifying changes that could be made to support implementation with little disruption to the practice workflow. A member of the research team takes structured notes that document the meeting attendees, agenda topics, and action steps.

#### Narrative reports

During the implementation phase, the practice facilitators conduct site visits to observe fidelity to the implementation process. During these visits the facilitators shadow practice staff and providers as patients move through the ALTA workflow and conduct informal interviews, developed for this study (see template for interview questions in additional file 2) to gather information about their experiences implementing the model. Practice facilitators capture notes from the visits on structured narrative reports.

#### Root cause analysis and PDSA worksheets

The practice facilitators complete structured root cause analysis and PDSA worksheets during site visits to the practices. The root cause analysis worksheet captures an issue that is identified as serving as a barrier to implementing an intervention component(s), the root cause of the issue(s) (e.g., possible reasons why the issue is happening), and possible action items to address the root cause (with information on what, how, when and with whom). The PDSA worksheet complements data captured on the root cause analysis by testing and documenting the effectiveness of the action items to mitigate the identified issue(s).

### Data management and analysis

Using the FRAME-IS modules as a starting point, the research team created an Excel spreadsheet that captures: (1) the specific implementation strategy and modification(s) being made; (2) modification category (e.g., changes to the content of the strategy or the way it is delivered); (3) nature of the modification (e.g., removing/adding/refining strategies); (4) goal of the modification and at what level (e.g., practice, provider, staff); (5) when the modification was made (pre-implementation, implementation) and whether it was planned; (6) who was involved in the decision; and (7) scope of the modification (e.g., one practice team vs. all participating practices). For our coding, we define *proactive* adaptations as changes to implementation strategies in response to anticipated barriers (e.g., a known change in practice staffing prior to implementation). Alternatively, *reactive* adaptations are changes to the implementation strategies due to unanticipated barriers during the implementation process (e.g., in response to a PDSA cycle). *Planned* adaptations are proactive or reactive changes that are decided using a systematic process that includes consulting the adoption metrics derived from EHR data (e.g., RPM orders, delivery of nurse RPM education, appointment scheduled for virtual clinic, transmission of home BP data), discussions with the implementation committee, and consideration of their impact on the implementation and intervention outcomes. *Unplanned* adaptations are changes made to implementation strategies without a systematic process (e.g., originate from practice staff and providers in response to implementation barriers without consulting implementation committee and/or data).

To facilitate tracking of our multifaceted PF strategy, the team also included fields to document the specific intervention component the implementation strategy targets (i.e., identify, refer, coach, document, monitor), a description of the original and modified implementation strategies using the ERIC taxonomy, and any overlap in the categorization of modifications for each strategy (e.g., what is being modified [module 2], the nature [module 3] and goal of the modification [module 4]). Categorization of the original and adapted implementation strategies using the ERIC taxonomy served two main purposes: (1) it facilitated discrete capture of the different implementation strategies used to support integration of ALTA into routine care and (2) allowed the team to determine whether changes to the strategies deviated from the core elements of PF (i.e., a metric of fidelity).

**Fig. 2:**
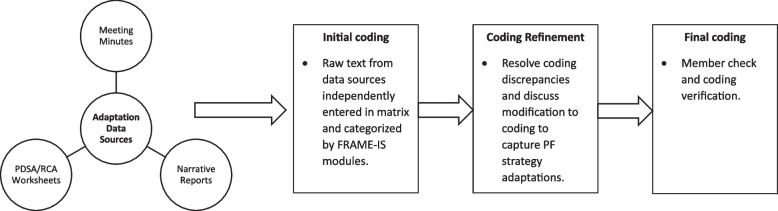
Process for coding and analyzing adaptation data

We followed best practices from previous research to code and categorize adaptations across the three data sources (Fig. 2) [24–26]. Text was extracted from the meeting minutes, narrative reports, and worksheets according to the FRAME-IS modules and inputted into a master matrix for content analysis. Two members of the research team independently entered the raw text data into the matrix from each source. A unique identifier was created for each document type (i.e., meeting minutes = MM; narrative reports = NR; Worksheets = WS, see additional file 1) to track the primary source of information as well as capture whether adaptations were represented by multiple data sources. After all data were entered into the matrix, the coders met to resolve any discrepancies in coding and discuss modifications to the FRAME-IS modules to adequately capture adaptations to the PF strategy. The research team also reviewed the matrix to identify similarities and differences in categorization of the adaptations across the data sources. Member checking was conducted by a third individual on the research team who reviewed the completed matrix, provided input on the proposed FRAME-IS modifications, and validated coding decisions.

## Results

### Goal 1: application of the FRAME-IS framework to a multifaceted PF strategy

We identified 27 adaptations across the pre-implementation and implementation phases, of which 24 (88.9%) were focused on implementation strategies to support the identify and refer components of the ALTA intervention. Most commonly, adaptations were identified through meetings of the implementation committee (41.7%), followed by narrative reports by the practice facilitators (30.6%) and notes on the root cause analysis and PDSA worksheets (27.8%). Below and in the supplemental table (additional file 1), we describe the adaptations based on the FRAME-IS modules as well as the unique categorizations that the research team created to account for the multifaceted PF strategy.

#### Modules 1–3: what was modified and nature of modification

Approximately half (52.9%) of the adaptations were made to the way the implementation strategy was delivered (i.e., context). Of these adaptations, about one third (36.3%) focused on changing the format of the implementation strategy from individual focused to group based. For example, the practice facilitators noted on the narrative reports that participating in morning huddles with the entire care team was a more effective strategy for creating practice goals to implement ALTA than targeting single providers during site visits. The remaining context adaptations focused on changes to who delivered the implementation strategy (personnel, 36.3%) as well as who was the target of the strategy (population, 27.2%). A notable change to personnel included the addition of community health workers (CHW) as core members of the PF strategy. The root cause analysis worksheets noted that patients were experiencing several significant technology-related barriers that were negatively impacting uptake of ALTA. With support of the implementation team, the CHWs were integrated into the PF strategy to provide one-on-one technical support to patients. The CHW tasks include assisting patients in finding, downloading, and using the BP apps on their phones and navigating the patient portal.

Another notable contextual adaptation was a shift in the population that was the primary target of the PF strategy. ALTA was originally conceptualized as a MA-led model, however, conversations with the FQHC leadership as well as policy-level considerations for reimbursement (i.e., the outer setting) led to nurses being the primary focus of ALTA. The remaining adaptations were categorized as training and content (17.6% and 29.4%, respectively). Training adaptations were mainly done to support asynchronous, self-paced learning of the intervention content (e.g., proper BP measurement). Content modifications included adding elements to the original PF strategy to support peer-to-peer learning and the technological infrastructure required to implement ALTA. As shown in the supplemental table (additional file 1), a comparison of the original and modified ERIC categories showed that none of the adaptations removed core elements of the PF strategies and were considered fidelity consistent.

#### Module 4: goals of the modification and level

Overall, 23 goals were listed across the multiple adaptations, of which 39.1% were made to increase the reach of ALTA to the patient population with the intent of reducing disparities in the delivery of the intervention. The higher prevalence of increasing reach as a goal aligns with more frequent adaptations made to the PF strategy to support the identify and referral components of ALTA. Examples of modified strategies to improve reach were tailoring materials so they are offered in multiple languages and expanding the home BP app capabilities to support iOS and android phones. Adaptations were also made to increase acceptability and adoption (13% each) by practice staff, improve fidelity in the intervention delivery, the fit with the practice workflow, and the clinical effectiveness and sustainability of ALTA (8.7% each). Approximately one-third (36.4%) of adaptations were made at the level of the organization (i.e., primary care practices) and the practitioner (i.e., FQHC providers and staff delivering ALTA). Fewer adaptations were made at the level of the patient and the implementors leading the PF strategy (13.6% each).

#### Module 5: when were the modifications made and were they planned

Except for one modification, all changes made to the PF strategy were planned. In most cases (84.6%), the modifications were reactive and occurred within the implementation phase in response to findings from the root cause analysis and PDSA cycles. The unplanned modification was led by practice nurses who developed a tracking system to identify eligible patients for ALTA and reduce missed opportunities for enrollment. While this adaptation was unplanned and reactive to their experiences implementing ALTA, this strategy was eventually spread to other practices due to its effectiveness in increasing enrollment.

#### Module 6–7: who participated in the decision to modify and how widespread were the modifications

Decisions about the modifications were mainly made by the FQHC leadership (40.9%) and members of the implementation committee (36.4%), which comprised both the FQHC and research teams. About one-quarter of the decisions also involved the FQHC providers and staff implementing ALTA (22.7%). Adaptations to implementation strategies were mainly widespread (83.9%) and shared across the network of participating practices. In some cases (16.1%), adaptations were localized to individual practices due to their unique implementation climate. For example, peer-to-peer learning among staff was an important strategy modification for practices that lacked a manager who could lead the implementation effort.

### Goal 2: considerations and lessons learned when using the FRAME-IS to characterize multidimensional implementation strategies in the context of a complex practice-based intervention

As shown in Table 1, we originally conceptualized the PF strategy supporting ALTA as distinct and focused on a singular ERIC category. In practice, the strategies are numerous and often interrelated making discrete data capture difficult when applying the FRAME-IS coding system. Moreover, it is often challenging to distinguish between what is within the scope of the PF strategy, which is inherently designed to be flexible, and what could be considered an adaptation. Despite these difficulties, there is great value in documenting the ongoing refinement of multifaceted implementation strategies that are needed to support implementation of interventions into real world practice. In this sense, we view the FRAME-IS as a helpful tool to track the ongoing optimization of our PF strategy to improve fit of the intervention to the practice context. To guide the documentation process, we considered an adaptation to have occurred if any of the following conditions were met: (1) the change in strategy was made in collaboration with the FQHC in response to practice-level implementation barriers; (2) the change represented a shift in focus from the originally planned strategy to optimize fit of the intervention; and/or (3) the change was initiated by practice providers or staff (i.e., unplanned).

To keep track of the multiple changes that occurred we also created a numbering system that allowed us to capture the breadth and depth of adaptations across the modules. Using this method, we created part 2 of module 2 (What is modified) to add a detailed list of the various modifications that were made to each PF strategy. For example, as shown in the supplemental table (additional file 1), rather than solely indicating the modification type as ‘training’, our matrix also included the specific adaptations to the PF strategy that fell within this category (e.g., using a train-the-trainer method, creating online training for asynchronous learning, etc.). Numbering each adaptation allowed the team to map the changes to the other modules and provide data on the most common ways strategies were modified, the different goals of each strategy, when they occurred, and how widespread.

## Discussion

In this paper, we describe the application of FRAME-IS to track adaptations to a PF implementation strategy. We identified over 20 adaptations to our multifaceted strategy, which were mainly focused on supporting practices in identifying patients with uncontrolled HTN who are non-adherent to their medications and referring them for RPM and health coaching for BP control. These adaptations were commonly made to attain the goal of enhancing the reach of our evidence-based intervention, ALTA. Over half of the adaptations were made to modify the context of the PF strategy to include a group-based format, add CHWs as new team members, and to shift the implementation target to nurses. The adaptations were often widespread, affecting all practices within the FQHC network. While nearly all the adaptations were reactive in nature, they resulted from a systematic (planned) process of reviewing data captured by multiple objective (EHR-derived) and subjective (narrative reports) sources. Finally, most of the adaptations to the PF strategy resulted from a discussion between the FQHC and research teams. To enhance intervention fit, it is equally important that the decisions to adapt strategies are made in partnership between the research and practice teams. Incorporating both perspectives into the adaptation process can help to align organizational resources, capabilities, and priorities of the practice while also facilitating rigorous evaluation of implementation outcomes [27].

Our paper adds two key contributions to the growing literature. First, this is one of the first papers to describe the application of the FRAME-IS modules to a PF implementation strategy. We initially experienced challenges in coding the multifaceted strategies that comprise PF using a singular code. For example, in several cases an adaptation could be characterized as a context change to both the format and personnel. Two previous studies described similar experiences when using the FRAME-IS to document adaptations to multifaceted implementation strategies supporting integration of practice-based interventions [13, 14]. For example, Mangale et al. [13] found that adaptations to the implementation strategies were not mutually exclusive and fit within several categories resulting in ‘unavoidable overlap.’ Haley et al. [9] also described challenges to tracking adaptations to a multifaceted implementation strategy using existing frameworks. Like our approach, the authors addressed these challenges by adding more detailed information on the changes using the ERIC taxonomy and developed new codes to augment the broad categorization of strategies offered by current frameworks. In our study, we also created a numbering system that allowed us to map the strategies across the FRAME-IS modules. Using this method, we were able to document each individual adaptation within a strategy as well as view the bigger picture of how the combination of adaptations supported implementation of ALTA. Moreover, use of the ERIC taxonomy to guide documentation of the original and modified implementation strategies enhances the generalizability of our work, as it supports transparency in identifying implementation strategy adaptations as well as the usage of common terminology to describe the strategies.

Second, this research provides key insights into the adaptations that are needed to support implementation of evidence-based interventions into safety-net settings. In this study, all adaptations were made in collaboration with the FQHC to further tailor the PF strategy to the inner (e.g., staffing models within each practice) and outer (e.g., reimbursement opportunities for health coaching led by nurse practitioners) practice setting. Interestingly, despite inclusion of a pre-implementation phase informed by the Consolidated Framework for Implementation Research that was designed to refine our PF approach, many of the adaptations were not identified until transitioning to the implementation phase. This may reflect the dynamic nature of safety-net settings who experience fluctuations in their staffing and resources (e.g., staff turnover) as well as hidden challenges that do not become apparent until the intervention begins, which in our case were technology-driven. Martinez et al. [15], also used the FRAME-IS to characterize adaptations of an integrated care model in FQHCs. Like our study, they found that adaptations were made to include practice providers and staff as key partners and to reflect service delivery changes experienced by the FQHCs, increase reach of the intervention, and leverage opportunities for reimbursement.

 This study also has several limitations. First, while the team captures adaptation data throughout the trial, the coding was completed retrospectively. This may have resulted in missed opportunities to capture real time changes in strategies over time. Second, most of our data was from qualitative reports completed by the practice facilitators, which may be subject to bias. Future data should include a greater diversity of data sources that combine qualitative and quantitative measures to capture adaptations to the implementation process. Finally, while we documented goals for each adaptation, we did not analyze these data to determine whether changes resulted in the intended implementation outcome (e.g., improving adoption of ALTA) because our trial is still ongoing. However, once our trial is complete, we will analyze these data to identify patterns in our adaptations and their impact on the effectiveness of our PF strategy on level of adoption and implementation fidelity (primary outcome) of ALTA.

## Conclusion

Applied examples of the FRAME-IS to document adaptations to multifaceted implementation strategies are needed to further refine the tool and increase its utility for diverse types of implementation science research. This study discusses our experiences using the FRAME-IS to document adaptations to a PF strategy that is supporting the implementation of an evidence-based technology facilitated team care model within a large FQHC. With some modifications, we demonstrate the ability to code our adaptation data across the FRAME-IS modules, attesting to its applicability and value for a range of implementation strategies.

Based on our experience, we offer the following recommendations for future research and practice. First, tracking systems need to evolve to allow for more nimble and practical documentation of iterative, ongoing, and multidimensional adaptations in real-time [1, 8]. While survey tools that embed branching logic can streamline documentation in support of these efforts, it remains essential that the number of questions and list of response options do not become too onerous for implementors to complete. Leveraging emerging best practices in natural language processing and machine learning is one avenue worthy of future exploration [28].

Future research would also benefit from tracking and reporting the intended and unintended process and clinical outcomes that result from the adaptation of implementation strategies. As argued by Kirk et al. [7], it is plausible that adaptations can create “unintended ripple effects,” whereby improvements in one outcome (improving fit) can decrease another (decreasing reach). Systematically tracking these data will improve our understanding of the most effective strategies that positively impact intervention outcomes. Finally, we advocate for extending Chambers and Norton’s [29] notion of an “adaptome,” a common data platform to store systematically captured intervention adaptations, to also include information on implementation strategies. This shared repository would help facilitate greater transparency in what constitutes a modification, the common ways strategies are modified and under what circumstances, and the downstream effects on implementation and interventions outcomes across multiple studies and contexts.

### Supplementary Information

Supplementary Material 1.

Supplementary Material 2.

## Data Availability

The datasets used and/or analyzed during the current study are available from the corresponding author on reasonable request.

## Abbreviations

ALTA: Advancing Medication Adherence for Long-term Improvements in Hypertension through a Team-based Care Approach
BP: Blood pressure
CHW: Community Health Worker
EHR: Electronic health record
ERIC: Expert Recommendations for Implementing Change
FQHC: Federally Qualified Health Center
FRAME-IS: Framework for Reporting Adaptations and Modifications for Implementation Strategies
HTN: Hypertension
PDSA: Plan Do Study Act
PF: Practice facilitation
RPM: Remote patient monitoring
