# Supplementary material for: Application of the FRAME-IS to a multifaceted implementation strategy
Source: BMC Health Serv Res. 2024 Jun 1;24:695. doi: 10.1186/s12913-024-11139-0 (PMC11143702; doi:10.1186/s12913-024-11139-0)
Supplement: Supplementary file 2 — Supplementary Material 2. [file 12913_2024_11139_MOESM2_ESM.docx]

| Additional File 2. Sample Narrative Report Form | | |
| --- | --- | --- |
| ALTA NARRATIVE REPORT FORM | | |
| **SITE: DATE: FACILITATOR:** | | |
| **Reason For Visit:** | Training  Investigate Issue (Box 1)  Meeting  Update  Observation  Other (Specify) |  |
| **Goal for Visit:** | \| **Did you meet your goal?** \| Yes  No (Explain Barriers) \| \| --- \| --- \| \| **Barriers to meeting goal:** \| \| |  |
| **Observations:**  ***Reflective Notes:***   1. ***How busy was the practice?*** 2. ***What are your overall thoughts on what you have observed?*** 3. ***What are key takeaways?*** | |  |
| **GUIDING INTERVIEW QUESTIONS AND OBSERVATIONS** | |  |
| 1. Describe what happens when a patient comes in for a visit. How does the MA? PCP/Nurse know a patient is ready to be seen? 2. Can you tell me the process you are using to take patients’ blood pressure?    1. When does a patient have blood pressure taken? How many times does the patient have their blood pressure taken? How long does the patient sit each time that blood pressure is taken? When is blood pressure recorded in the EHR? 3. Describe the actions you take after a patient had a high blood pressure reading.    1. Does the clinical staff discuss a patient’s blood pressure with the patient? What about with another member of the medical team?    2. What is discussed? 4. Tell me about how you ask patients about medication adherence.    1. Do they use the ALTA structured questions?    2. How long does that take? 5. Can you describe how different members of a patient’s medical team communicate with each other (I.e. secure messaging, in person) about ALTA?    1. What is the flow of communication? Is it verbal, via the EHR, etc.?      1. Did any patients accept ALTA and remote patient monitoring (RPM) during the visit that you were observing?    1. Which member(s) of the medical team talked to the patient about RPM?    2. Who did what?    3. What went well? What needs improvement? 2. Can you tell me about the process of onboarding patients onto RPM?    1. How long did the onboarding process take?    2. Who did what?    3. What went well? What needs improvement?    4. What information was communicated to the patient about next steps? | |  |
